# Biomimetic surface structures in steel fabricated with femtosecond laser pulses: influence of laser rescanning on morphology and wettability

**DOI:** 10.3762/bjnano.9.262

**Published:** 2018-11-05

**Authors:** Camilo Florian Baron, Alexandros Mimidis, Daniel Puerto, Evangelos Skoulas, Emmanuel Stratakis, Javier Solis, Jan Siegel

**Affiliations:** 1 Laser Processing Group, Instituto de Óptica, IO-CSIC, Serrano 121, 28006 Madrid, Spain; 2 Institute of Electronic Structure and Laser (IESL), Foundation for Research and Technology (FORTH), N. Plastira 100, Vassilika Vouton, 70013, Heraklion, Crete, Greece

**Keywords:** biomimetics, femtosecond laser irradiation, laser-induced periodic surface structures, laser rescanning, steel, wettability

## Abstract

The replication of complex structures found in nature represents an enormous challenge even for advanced fabrication techniques, such as laser processing. For certain applications, not only the surface topography needs to be mimicked, but often also a specific function of the structure. An alternative approach to laser direct writing of complex structures is the generation of laser-induced periodic surface structures (LIPSS), which is based on directed self-organization of the material and allows fabrication of specific micro- and nanostructures over extended areas. In this work, we exploit this approach to fabricate complex biomimetic structures on the surface of steel 1.7131 formed upon irradiation with high repetition rate femtosecond laser pulses. In particular, the fabricated structures show similarities to the skin of certain reptiles and integument of insects. Different irradiation parameters are investigated to produce the desired structures, including laser repetition rate and laser fluence, paying special attention to the influence of the number of times the same area is rescanned with the laser. The latter parameter is identified to be crucial for controlling the morphology and size of specific structures. As an example for the functionality of the structures, we have chosen the surface wettability and studied its dependence on the laser processing parameters. Contact angle measurements of water drops placed on the surface reveal that a wide range of angles can be accessed by selecting the appropriate irradiation parameters, highlighting also here the prominent role of the number of scans.

## Introduction

Complex structures found in nature often present properties that are attractive for applications in science and technology. The hydrophobicity found at the lotus leaf surface [1], the exceptional adhesion capability of gecko feet [2], or the colorful optical effects produced by the wings of a butterfly [3] are just a few examples of the many properties that have been successfully mimicked and used in different technological applications [4]. This area of science is called biomimetics, where many disciplines team up with the objective to reproduce not only the geometry and morphology of structures found in natural systems, but – most importantly – their specific functionality. Biomimetic applications that aim to control the wetting properties of a material surface must take into account the surface topography, since it strongly influences the surface energy and thus the wetting behavior [5–10].

A particular kind of controllable surface modification induced by pulsed lasers was discovered in 1965 by Milton Birnbaum [11] – upon irradiation of a germanium wafer with multiple laser pulses, self-organized periodic surface ripples were formed, featuring a period close to that of the laser wavelength with an orientation perpendicular to the laser polarization. This discovery opened a new field of research, and soon thereafter, similar and more complex self-organized structures were reported for many other types of materials [12–14]. Such structures are commonly referred to as laser-induced periodic surface structures (LIPSSs). Two main mechanisms have been proposed to explain the origin of these structures. One of them takes into account laser light scattered at a rough surface, which interferes with the incident pulse [15]. The other mechanism involves the formation of a surface plasmon polariton coupled to the sample–air interface, which interferes with the incoming pulse [16]. For both mechanisms, interference leads to a spatial modulation of the intensity distribution that is finally imprinted in the material by a variety of processes, including local ablation [17], amorphization [18], convection [19] and others. The type of structures that are formed is diverse and depends on the irradiation parameters, the most common ones being the so-called ripples (parallel lines with a period near the laser wavelength), grooves (parallel lines with supra-wavelength period), and spikes (supra-wavelength cone-like structures) [20]. As many studies show, LIPSSs can be fabricated without the need of highly focused beams at industrially relevant speeds [6,21–22] and are interesting candidates for controlling the wetting and friction properties of a material for numerous applications [5–6,8,23–24]. Yet the type of LIPSSs investigated so far for these applications was mostly limited to those mentioned above (ripples, groves and spikes), and less so the more complex structures that are accessible by exploring a broader range of laser processing parameters.

In this article, we demonstrate the strong influence of the number of times the area is rescanned with the laser on the type of structures that can be fabricated. This parameter has been seldom investigated [25–27] despite its strong influence on the surface morphology. We present experimental results of complex self-organized structures produced in commercial steel that resemble the morphology of the skin of certain reptiles and insects, which are of great interest due to their exceptional fluid transport and friction reduction properties. Surface characterization is performed via optical and scanning electron microscopy. Wetting measurements on the fabricated structures are presented, demonstrating that different wetting regimes can be achieved by a proper choice of the processing parameters, leading to the formation of hydrophilic, hydrophobic and superhydrophobic states.

## Results and Discussion

### Ripples, grooves and spikes formed by single laser scanning

**Figure 1:**
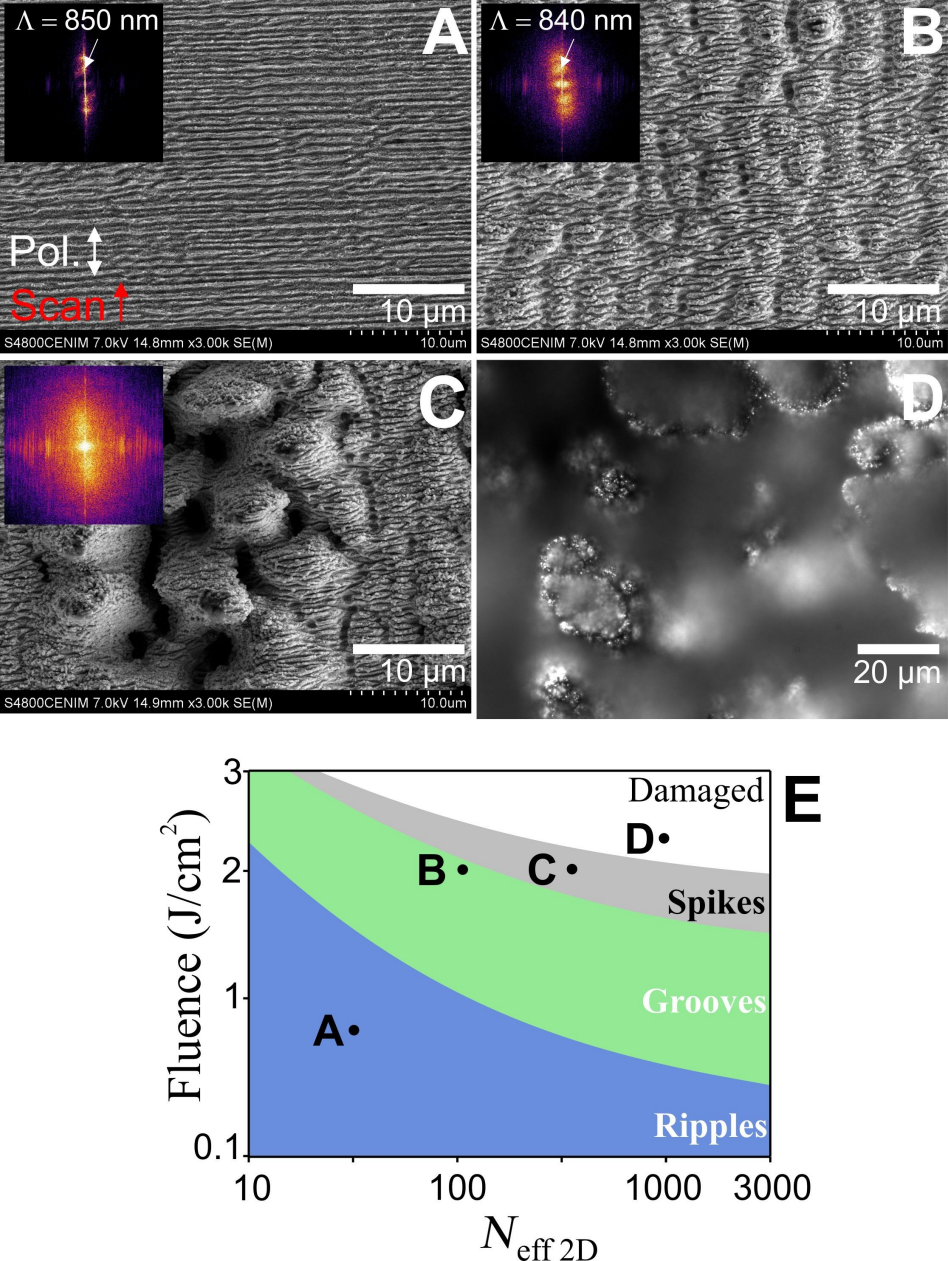
SEM images of the three LIPSS structures produced in steel under different laser irradiation conditions, distinguished fundamentally by the laser fluence and pulse number *N*_eff_2D_ (parameters given in the text): A) ripples with a periodicity Λ_y_ = 850 nm, B) grooves, Λ_y_ = 840 nm, Λ_x_ = 2.6 µm, and C) spikes. D) Highly irregular (“damaged”) morphology (optical micrograph) obtained at high fluence and *N*_eff_2D_ values. E) Schematic distribution of the different structures found with one single scan, depending on the laser fluence and the effective number of pulses in an area (*N*_eff_2D_). The positions on this plot for the structures shown in A–D are represented accordingly. The scanning direction and laser polarization for all the images shown are included in A.

The formation of LIPSSs occurs when a certain number of laser pulses has accumulated in a given area in a single laser scan. For metals and semiconductors, three well-differentiated structures, ripples, grooves, and spikes, can typically be formed either by increasing the effective number of pulses (*N*_eff_) for a given fluence 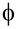 or by increasing the laser fluence for a given value of *N*_eff_ [19]. In our particular case, these three LIPSS types can be formed using a combination of parameters that depends on the geometry of the laser beam at the focus, the effective number of pulses *N*_eff_2D_ (given by the laser repetition rate ν, the scanning speed *V*, line separation Δ, c.f. Experimental section) and the laser fluence 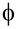 used. As shown in Figure 1A, ripples can be fabricated at 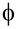 = 0.5 J/cm^2^ and *N*_eff_2D_ = 30. The morphology corresponds to horizontal parallel lines that are oriented perpendicular to the laser polarization. The periodicity imprinted into the material is Λ = 850 nm, measured from a fast Fourier transform (FFT) of the SEM micrograph, which is much smaller than the laser spot size (2ω_0_ = 38.8 µm), confirming the self-organizing nature of the formation process. Figure 1B shows grooves (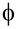 = 2 J/cm^2^, *N*_eff_2D_ = 117), for which horizontal ripples can still be observed but are superimposed by vertical grooves, oriented parallel to the laser polarization. Figure 1C shows spikes (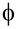 = 2 J/cm^2^, *N*_eff_2D_ = 407), which are self-organized cone structures distributed over the irradiated surface. A higher fluence with a higher number of pulses evolves into nonuniform structures without any visible order, indicating an excessive energy dose and resulting in severe damage of the material (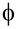 = 2.3 J/cm^2^, *N*_eff_2D_ = 1000, as shown in Figure 1D). A schematic distribution of the structures that can be obtained by performing a single laser scan, taking into account the laser fluence and the effective number of pulses *N*_eff_2D_, is included in Figure 1E. According to this scheme, the fabrication strategy with one single scan seems to be limited to these three relatively simple structures.

### Complex morphologies formed by laser rescanning

A viable method to increase the effective number of laser pulses up to hundreds of thousands of laser pulses, while preserving the formation of self-ordered structures, is scanning the laser beam many times over the same sample area, i.e., laser rescanning. From a physical point of view, one fundamental difference between a single scan and multiple rescans is that the incident laser pulse during the second and subsequent scans “sees” not only a higher surface roughness, but in many cases, ordered structures (namely ripples, grooves or spikes). The presence of these structures greatly influences the spatial distribution of the scattered light [26,28] and the coupling efficiency and propagation of surface plasmon polaritons. As a consequence, the spatial intensity distribution that leads to ablation changes strongly between scans. Moreover, for a constant total number of pulses and processing time [25], the thermal heating of the sample during a single scan is much larger compared to multiple rescans, for which the sample has time to cool between scans.

We have performed a systematic study of the influence of the number of rescans *n* on the structures formed. To this end, we have kept the effective pulse number *N*_eff_3D_ constant, distributing it into a different number of scans, according to *N*_eff_3D_ = *n* * *N*_eff_2D_, with *n* = 1, 100, 200 and 300 scans. Importantly, different scanning speeds have been used in order to ensure that the total fabrication time for a certain area remains constant, regardless the conditions used. In our case, processing of the 1 cm^2^ areas took 1 hour each. However, for specific applications, this time can be reduced considerably with the right combination of a high repetition rate laser and fast scanning head. The specific experimental conditions used to fabricate the different areas are displayed in Table 1, including 4 different laser fluence values.

**Table 1:** Experimental conditions used for the laser rescanning experiments. Please note that the number of total pulses (*N*_eff_3D_) corresponds to the effective amount of pulses delivered in an area equal to a spot area, not to the total number of pulses delivered in an area.

Fluence (J/cm^2^)	Rep. rate (kHz)	*n* = 1 scan (0.002 m/s)	*n* = 100 scans (0.2 m/s)	*n* = 200 scans (0.4 m/s)	*n* = 300 scans (0.6 m/s)

0.12	2000	*N*_eff_3D_ = 150000 pulses			
0.2	1000	*N*_eff_3D_ = 74000 pulses			
0.5	1000	*N*_eff_3D_ = 74000 pulses			
1	500	*N*_eff_3D_ = 37000 pulses			

**Figure 2:**
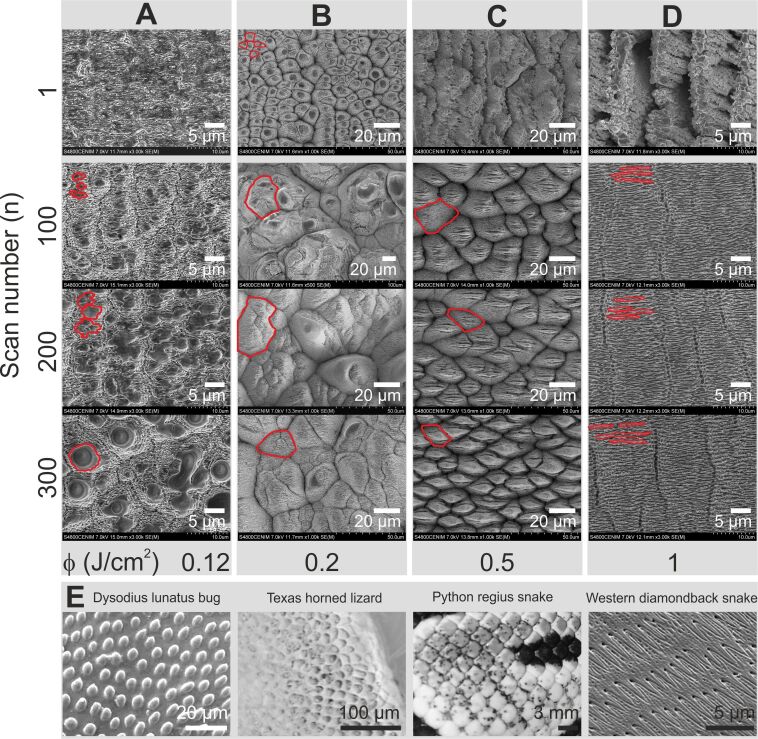
A–D) SEM images of the biomimetic structures fabricated in steel, with the experimental parameters given in Table 1, arranged according to increasing laser fluence 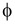 and number of scans. The total fabrication time for all the areas was kept constant by adjusting the scan speed and repetition rate accordingly. For all structure types, the characteristic shape of one or a few of the unit structures is outlined in red. In E), from left to right, images of the corresponding real structures found in the *Dysodius lunatus* bark bug [29], Texas horned lizard (*Phrynosoma cornutum*) (image adapted from [30], an article under a Creative Commons Attribution 2.0 License, http://creativecommons.org/licenses/by/2.0, copyright 2011 P. Comanns et al., i.e. the authors of [30]), *Python regius* snake (adapted from https://pixabay.com/es/snake-pit%C3%B3n-bola-python-regius-605344/), and Western Diamondback rattlesnake (image adapted from [31], an article under a Creative Commons Attribution 4.0 International License, http://creativecommons.org/licenses/by/4.0, copyright A. E. Filippov and S. N. Gorb).

SEM micrographs of the 16 different areas corresponding to the experimental conditions shown in Table 1 are displayed in Figure 2. The images demonstrate that laser rescanning indeed allows processing the sample with extraordinarily high *N*_eff_3D_ values and still generates self-organized structures whose morphology is not limited to the aforementioned simple structures. At these high *N*_eff_3D_ values, the morphology obtained by a single scan (*n* = 1) leads to the formation of morphologies that are drastically different from those obtained by rescanning the same area of the sample, especially at high fluence values.

In particular, Figure 2A shows the results obtained at the lowest fluence, 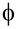 = 0.12 J/cm^2^. For a single scan (*n* = 1), a rather flat morphology with randomly distributed small particles is observed. The absence of any of the typical self-organized structures (ripples, grooves and spikes) can be understood by the extreme situation of a very slow speed (*V* = 0.002 m/s) and high repetition rate, leading to high heat accumulation [32–33]. Rescanning the sample area *n* times while maintaining a constant total number of pulses *N*_eff_3D_, leads to the formation of tiny cone-like structures of increasing diameter with the number of rescans. The smooth tips visible in the SEM images correspond to protrusions, whereas the rougher regions are located at the bottom. Ling et al. [26] and Zhulke et al. [27] provided a convincing explanation for the formation of similar cone-like structures, which were observed at similar fluences and also high pulse numbers, but with low repetition rate lasers (1–10 kHz) and without keeping the total pulse number constant. The authors showed that, in a first stage, small precursor cones are formed at impurities of the sample. In a second stage, the conical geometry itself, featuring slopes that increase the Fresnel reflection losses of the incident laser light and that have a larger area compared to a corresponding flat surface, leads to a significant reduction of the absorbed fluence. As a consequence, the area surrounding the cone is ablated at a higher rate than the cone. It has to be said, though, that the tips of our cone-like structures are much smoother than those reported in [26–27], which can possibly be attributed to heat accumulation at the high repetition rates used.

From a biomimetic point of view, this type of structure resembles the integument of bark or flat bugs, found in South and Central American tropics [23,34], as illustrated in Figure 2E. The function of the bug’s micro- and nanostructures is to allow rapid water transport all over its cuticle, which serves as camouflage during rain in their natural environment, changing the bug’s color to make it indistinguishable from the bark of some trees it rests on. Laser-based self-organization experiments in steel exploiting spike structure formation upon a single laser scan have been reported in [23], showing good performance for fluid transport applications but less similarity in morphology to the bug cuticle.

The results obtained at 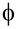 = 0.2 J/cm^2^ are shown in Figure 2B. For a single scan at such high *N*_eff_3D_ values, the morphology corresponds to randomly oriented but well-defined cone structures. To the best of our knowledge, such well-defined cone structures have not been reported before for a single scan, which is likely related to the high pulse number used in our case and underlines the interest of such high values for the fabrication of new surface morphologies based on self-organization mechanisms. Like for the structures observed at lower fluence, the cones have a smooth tip that is possibly related to heat accumulation. For rescanning at *n* = 100, similar structures appear, although at a much larger spatial scale, approximately five times wider. These cone structures also bear similarities to those reported in [27] and their formation is most likely also influenced by reduced ablation of the cones, formed at imperfections. As can be seen in Figure 2B, the size of the structures decreases somewhat upon increasing number of scans, although still well above the initial size of the cones for a single scan.

Within a biomimetic context, similar structures can be found on the skin of the Texas horned lizard, as shown in Figure 2E [30]. Their function is also related to water transport, although in this case not for camouflage but hydration purposes, which is vital considering the natural habitat of this lizard is the desert. The humidity in the air or moisture at the surface of rocks on which the lizard is standing can condense at the skin tiles, flowing towards its mouth where it can drink it actively. Recent studies based on direct laser writing have demonstrated that is possible to mimic the lizard skin tiles and micro–nano-ornamentation in different materials to successfully obtain directional liquid flow [30,35]. The present results show a new route for mimicking this structure by exploiting self-organization mechanisms. In particular, employing a combination of such isotropic microstructures shown in Figure 2B (which determine the water contact angle, as will be shown in Figure 4) with an anisotropic overstructure with dimensions of hundreds of micrometers (which leads to directionality) can effectively produce preferential fluid transport. Moreover, the results shown in Figure 2B suggest that the cone size can be conveniently adjusted by a proper choice of the number of scans.

At higher fluence (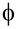 = 0.5 J/cm^2^), the morphologies obtained are dramatically different. Figure 2C shows that for a single scan, no apparent order or self-organization is obtained. Upon rescanning, ordered cone-like microstructures are formed whose size decreases with the scan number. It is questionable though if at this relatively high fluence the scenario of cone formation triggered at the defects remains valid. An interesting ripple-like substructure can be observed in the center of the cones, whose orientation is consistent with the ripples structures formed upon irradiation with a low number of pulses (Figure 1A) although the ripple width is much larger and not well defined.

In terms of biomimetics, these structures resemble the tiles found on the skin of the *Python regius* snake, whose microstructure makes it very resistant to damage from wear by reducing friction (c.f. Figure 2E). Laser-based surface texturing has been used to mimic this structure in steel [36], demonstrating considerable friction reduction although with only limited morphological similarity. Our approach based on laser-induced self-organization upon rescanning leads to a better similarity, and the functional performance in terms of wettability shows extraordinary results, as will be shown in a later part of the paper.

The structures fabricated at the highest fluence, 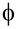 = 1 J/cm^2^, are shown in Figure 2D. As before, for a single scan no apparent order is observed, whereas rescanning leads to the formation of self-organized structures with a large degree of order, specifically horizontal ripple structures with signs of vertical grooves that are much wider than the ones shown in Figure 1B for single scan experiments at low fluence and low pulse number. The overall morphology is similar to that found on the skin of the Western diamondback rattlesnake (Figure 2E [31]). In a recent study, structures with similar geometry have been mimicked on titanium substrates with the aim of demonstrating friction reduction [37]. In our case, we not only manage to mimic the shape but also their characteristic micrometer size, which may play an important role on the performance for real friction reduction applications.

Overall, the results obtained with rescanning and very high pulse numbers demonstrate a high versatility for the fabrication of structures with very different morphologies, all based on self-organization. As can be seen in Figure 2, the structure sizes range from 800 nm up to 50 µm, with the nanostructures located generally at the bottom of the formed structures.

### Depth and density of the structures

**Figure 3:**
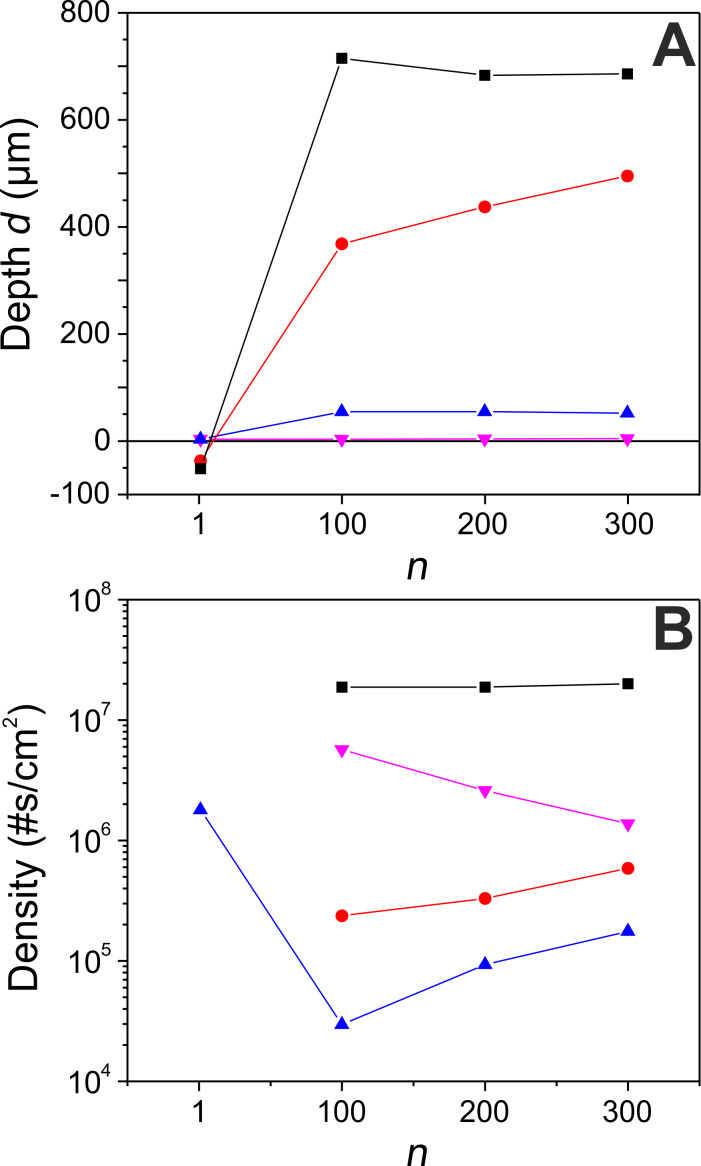
Evolution of the depth *d* of the structured fields (A), and the density of structural units (B) produced vs the number of rescans, *n*. The different curves correspond to different experimental conditions, as indicated in Table 1. The plotted fluences are 0.12 (pink triangle), 0.2 (blue triangle), 0.5 (red circle) and 1 J/cm^2^ (black square).

The depth of the structured fields was measured with an optical microscope and the results are summarized in Figure 3. It is important to keep in mind that the total number of *N*_eff_3D_ was kept constant for a given fluence. For the lowest fluence (0.12 J/cm^2^), only a very small amount of material is ablated, independent of the number of rescans (less than 2 µm, in the range of the microscope resolution for the *z*-axis). It should be noted that in this case the fluence is slightly below the single pulse ablation threshold, which we have determined experimentally as *F*_abl_ = 164 mJ/cm^2^ (following the method proposed by Liu in [38]). This indicates that the ablation is mediated by incubation and/or heat accumulation at the high repetition rate used [39–40] (a more detailed investigation of this process is out of scope of the present study). When the fluence is increased to 0.2 J/cm^2^, the ablation depth remains very small for a single scan but increases considerably upon rescanning, reaching a constant value of *d* = 50 µm. In view of the same incident pulse number and relatively similar morphology of single scan and rescanned structures (Figure 2B), differing mainly in the lateral dimensions of the cones, it is likely that the small depth value for a single scan is caused by the difficulty to resolve the depth between cones due to their narrow spacing. For the fluence values of 0.5 J/cm^2^ and 1 J/cm^2^, an interesting phenomenon is observed. Here, a single scan actually leads to negative values in the plot in Figure 3, which corresponds to a material protrusion above the initial material surface over the entire processed field. We attribute this phenomenon to the formation of a porous structure in combination with oxidation, mediated by the high temperature reached over a long time, as a consequence of the slow scan speed and high fluence. This interpretation is supported by the brownish color of the surface that can be realized upon inspection. Somewhat similar results have been reported in [41–43], showing a laser-induced decrease of material density via the creation of cavities, obtaining a foam-like structure. This phenomenon is not observed for laser rescanning, reaching ablation depths as high as 700 µm for the highest fluence and highest number of rescans. Interestingly, the morphology of these very deep fields is rather smooth, which holds promise for low roughness material processing applications.

Moreover, we have analyzed the SEM images shown in Figure 2 in terms of number density of structure units using the outlining of a structural unit given in the same figure as a criterion. The results are shown in Figure 3B, displaying the density of units per cm^2^ as a function of scan number *n* for the four fluence values studied. It can immediately be appreciated that the density is strongly fluence-dependent, ranging over almost three orders of magnitude from 3 × 10^4^ cm^−1^ to 2 × 10^7^ cm^−1^. In contrast, for a given fluence, the evolution of the density with scan number is much smoother and monotonous (except for 0.2 J/cm^2^, which is the only case of a new self-organized structure upon single scan). These results illustrate the potential of this technique for the fabrication of biomimetic structures of different shapes and at a widely variable density that can be fine-tuned to some extent by varying the scan number.

### Wetting characterization

The wettability of a material surface is an essential property that can define the range of applications it can be used for. For rough surfaces, an important question is whether the liquid on top completely fills up the roughness grooves (homogeneous wetting regime) or leaves air inclusions entrapped inside the grooves (homogeneous wetting regime), which strongly affects the apparent contact angle [44–45]. For these two regimes, also termed Wenzel and Cassie-Baxter regimes, respectively, wetting can in principle be described by taking into account the interfacial tension between substrate, liquid, and vapor and the surface geometry. Yet, the sole presence of one or another regime is a matter of debate [45]. While this dependence of surface wetting on surface energy and topography makes it particularly difficult to predict the wetting scenario, knowledge of the apparent contact angle alone is often sufficient for practical applications.

For the particular case of steel, it is known that the laser-processed material evolves from an initial superhydrophilic state to a (super-) hydrophobic state within approximately 10 days, due to the progressive attachment of carbon and its compounds to the surface [46]. After this time, the surface has stabilized chemically and also in terms of its wetting behavior. As explained in the Experimental section, the surface wetting was measured after at least 15 days after irradiation so that the surface was stabilized, allowing the formation of this free low energy coating required for a stable surface.

**Figure 4:**
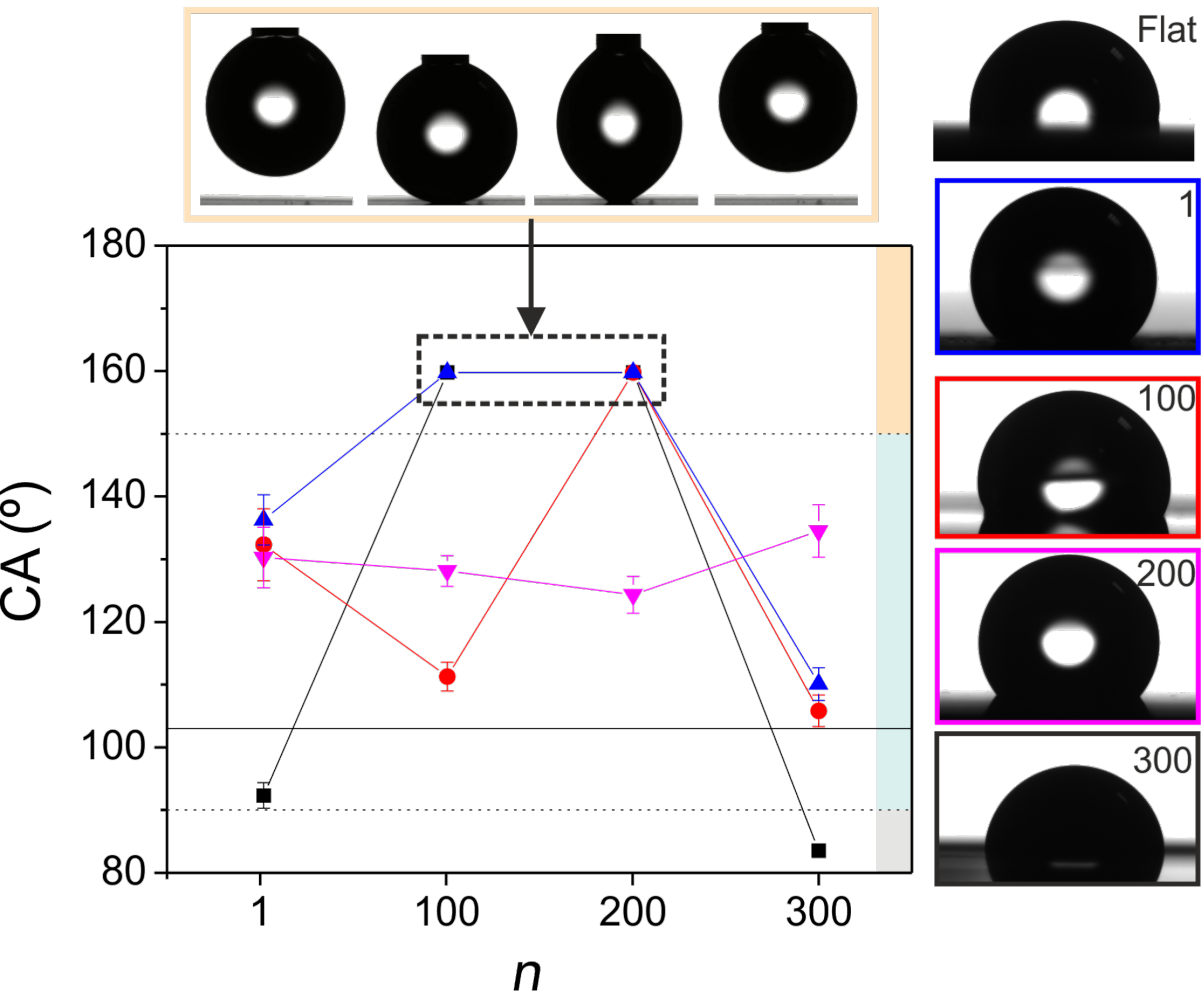
Side view of water droplets placed on different laser-processed areas. The labels/frame color of the right column of micrographs indicate the specific scan number and fluence used. The experimental conditions for each case are available in Table 1. The plot shows the evolution of the contact angle (CA) as a function of scan number *n*. The plotted fluences are 0.12 (pink triangle), 0.2 (blue triangle), 0.5 (red circle) and 1 J/cm^2^ (black square). Points plotted with CA values above 150° correspond to superhydrophobic surfaces that did not allow the positioning of the droplet (no CA could be measured in those cases). The top row of micrograph illustrates a time sequence of such a case, summarized in four stages: approaching, contact, attempt to retrieve the syringe, unwanted removal of the drop. A video is included as Supporting Information File 1 where can be seen that after several attempts the water droplet does not land on the laser irradiated surface. A color code is included at the right part of the plot in order to distinguish between surfaces with different wetting behaviors. Superhydrophobic surfaces in yellow (CA > 150°), hydrophobic surfaces in blue (150° > CA > 90°) and hydrophilic surfaces in grey (90° > CA > 10°). The CA of untreated steel is 103° indicated by a solid line.

A straightforward way to measure the apparent contact angle wetting behavior of a surface is to deposit a small droplet of liquid on top of it and measure the contact angle (CA) formed between the substrate and the contour of the droplet from a grazing viewing position. Figure 4 includes representative images of those measurements in combination with a plot of the measured CAs on all fabricated areas. The measured CA of the unprocessed sample is included in the plot as a solid line at 103 ± 2°, with an inset labeled as “flat”. When the CA lies between 10° and 90°, the surface is considered hydrophilic, which is the case for the area produced at 1 J/cm^2^ and 300 rescans. For CAs between 90° and 150°, the surface is considered hydrophobic. In our experiments, more than half the fabricated surfaces present this wetting behavior, changing from 92 ± 2° (1 J/cm^2^, 1 scan) to 136 ± 6° (0.2 J/cm^2^, 1 scan). Surfaces that present CAs higher than 150° are considered superhydrophobic. In our case, data plotted over 150° correspond to areas where the droplet did not land on the surface, even after several attempts, so the real angles could not be measured, as shown in the inset on these CAs and in the video included as Supporting Information File 1. From the plot shown in Figure 4, it can be concluded that for the lowest fluence (0.12 J/cm^2^) the contact angle can be increased by 25° with respect to the unprocessed surface, irrespectively of the number of scans. In contrast, for higher fluence values, the CA depends strongly on the number of scans and CAs both higher and lower than the unprocessed surface can be obtained.

It is worth pointing out that one possible additional reason for the pronounced differences in the CA for a given fluence is the different heat accumulation generated in the material during laser processing. As indicated in Table 1, the use of a constant *N*_eff_3D_ leads to different scan speeds and thus different heat accumulation [33,47].

## Conclusion

We have demonstrated that scanning the laser beam many times over a steel surface (i.e., laser rescanning) leads to morphology changes that are very different from those produced with the conventional single scan approach, even when keeping the total number of pulses constant. In particular, the obtained structures resemble those found on the skin of the Texas horned lizard, the *Python regius* snake, the western diamondback rattlesnake, as well as on the integument of the bark bug, featuring fluid transport and friction reduction capabilities. Characterization of the wettability of the structures revealed that a broad range of different wetting behaviors could be accessed with this approach, ranging from hydrophilic over hydrophobic to superhydrophobic states, with practically the same fabrication time. It is found that the scanning speed in combination with the total number of rescans has a strong influence on the final morphology of the sample in terms of structure size and field depth. The fabricated structures show potential for use in applications for fluid transport and friction reduction applications.

## Experimental

**Figure 5:**
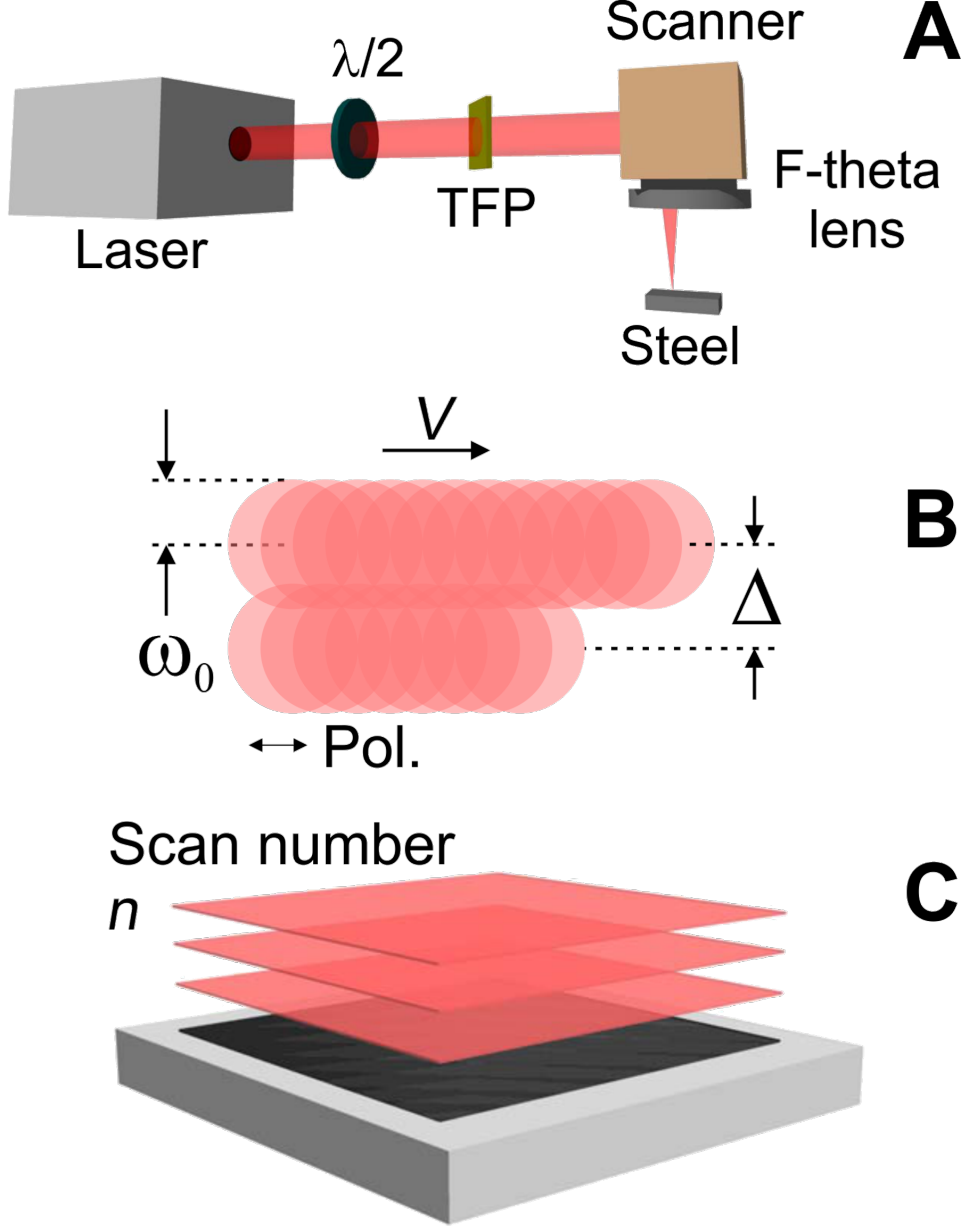
A) Experimental setup, employing a high repetition rate femtosecond laser system and a beam scanning system. B) Continuous irradiated areas are formed by overlapping consecutive laser pulses (ω_0_ = 19.4 µm) in two dimensions through a proper adjustment of the laser repetition rate (ν), the scanning speed (*V*), and the line separation (Δ). C) Several continuous areas are stacked by rescanning the same area *n* times.

The laser system used for the experiments was a femtosecond Yb-doped fiber laser from Amplitude Systems with an adjustable repetition rate (ranging from 1 kHz to 2 MHz), maximum output power of 40 W, laser wavelength of 1030 nm and a pulse duration of 340 fs. An F-theta lens with focal length of 100 mm was used to focus the laser beam down to a spot with Gaussian intensity distribution and a diameter 2ω_0_ = 38.8 µm. The laser system is computer-controlled and synchronized with a galvanometer-based mirror scanner positioned before the lens, which allows scanning the laser spot over a field as large as 70 × 70 mm^2^ at speeds up to 7 m/s. The static samples were mounted on a 3D mechanical stage provided with micrometers to position the sample perpendicular to the beam axis. A sketch that contains the main parts of the setup is displayed in Figure 5A.

The sample used for the experiments was commercially available 1.7131 steel (also termed 16MnCr5: C – 0.14 to 0.19%, Si – 0.4% max., Mn – 1 to 1.3%, P – 0.025% max., S – 0.035% max. and Cr – 0.8 to 1.1%) supplied by Miba Gleitlager GmbH (Laakirchen, Austria). The samples were cut to a lateral dimension of 45 × 45 mm^2^ with a thickness of 0.5 mm. The surfaces were polished obtaining mirror-like quality with an average roughness *R*_a_ < 2 nm measured by an atomic force microscope (AFM, Agilent 5100 AFM/SPM in tapping mode). In order to avoid environmental oxidation by humidity, the samples were stored in a desiccator at 30% relative humidity. Before and after laser irradiation, the samples were cleaned in an ultrasound bath with isopropanol for 5 minutes and gently dried with pressurized nitrogen.

The irradiation strategy consisted of fabricating continuous areas of 5 × 5 mm^2^ with an adequate balance between the laser repetition rate (ν), scan velocity (*V*) and interline distance (Δ), which determine the effective number of pulses given by *N*_eff_2D_ = 
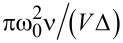
, as shown schematically in Figure 5B. The number of consecutive rescans (*n*) was 1, 100, 200 or 300, illustrated in Figure 5C as vertically stacked areas. The effective number of pulses in this three dimensional configuration corresponds to *N*_eff_3D_ = *n* × *N*_eff_2D_. In order to minimize the deposition of debris, a fume extractor was placed near the irradiation area. All irradiation steps were performed in air.

Optical inspection of the structures and depth measurements of the fabricated areas were performed with an optical microscope (Nikon Eclipse-Ti) using 460 nm illumination and objectives with numerical aperture of 0.06, 0.3 or 0.9 and nominal magnification of 2.5×, 10× or 100×. The nominal maximum lateral resolution of the system is *R*_xy_ ≈ 255 nm and *R*_z_ ≈ 1.1 µm in the vertical direction. The microscope was equipped with a motorized stage to allow focusing–defocusing, which allowed an estimation of the structure depth by focusing on the non-irradiated surface and comparing the *z* position at this point with the position when the focus is on the bottom of the irradiated area. In most of the cases, the depth was positive (the laser ablated material producing a hole in the sample) whereas in some cases, negative depths where also measured (the irradiated sample protrudes out). In addition, the morphology of the surfaces was characterized with a field emission electron microscope (Hitachi S-4800).

The instrumentation used to measure the contact angle (CA) was an OCA 15EC system that allows mechanical deposition of 4 µL deionized water droplets on the areas of interest. The system has a homogeneous illumination source and imaging optics that project the image of the droplet onto a CCD camera, capturing snapshots or recording videos that are later analyzed by dedicated software. The samples were characterized at least 15 days after irradiation to allow surface stabilization, since it has been demonstrated that irradiated areas of steel measured in less than 10 days present significant CA changes [46]. The errors specified for the different CA values were obtained via software analysis.

## Supporting Information

File 1 Wettability test of a laser-structured area, exposed to a laser fluence of 1 J/cm^2^, 200 rescans and a repetition rate of 500 kHz. The water droplet touches the surface several times, but never lands due to the high hydrophobicity of the structured area.
